# Hospital and Institutional News

**Published:** 1916-10-07

**Authors:** 


### HOSPITAL AND INSTITUTIONAL NEWS.

### THE QUEEN AT THE ROYAL FREE HOSPITAL.

The Queen visited the Royal Free Hospital, Gray's Inn Road, on October 2, for the purpose of opening the extensions of the London School of Medicine for Women. Her Majesty was received at the main entrance by Miss Aldrich Blake, M.D., Dean of the school; the Duchess of Marlborough, honorary treasurer of the Extension Fund; Mr. F. D. Acland, M.P., chairman of the Council; and Miss L. M. Brooks, secretary and warden. In the Common-room were representatives of the Council, the staff, and the students. At the ceremony itself, in what will be the anatomical department of the school, were also present the Chancellor of London University, Lord Rosebery, and the Vice-Chancellor, Sir Alfred Pearce Gould; the Presidents of the Royal Colleges of Physicians and Surgeons. After speeches by the Dean and others, the Queen formally declared the building open, and then went through the anatomy laboratories, the physiology laboratories dedicated to the late Miss Julia Cook, as well as those devoted to organic chemistry, physics, and pathological research. At the present time, it is stated, the students number 380; a very large entry has been received for the new session, which is also the experience of all the schools of medicine where women are accepted as students.

### THE NEW HOSPITAL FOR WOMEN, EUSTON ROAD.

A few days ago the testamentary dispositions of the well-known woman writer, Miss S. B. MacNaughtan were published, and £1,000 was bequeathed by her to the New Hospital for Women. This institution, or rather the small dispensary which afterwards grew into the present hospital, was founded exactly fifty years ago by Dr. Louisa Garrett, better known as Mrs. Garrett Anderson, in Lower Seymour Street, Edgware Road. The house is still in existence, though it is stated to be in a very dilapidated condition. To commemorate the jubilee of the New Hospital the committee have instituted a series of lectures, to be given in girls' schools and colleges of higher education. The first lecture was given about a week ago by Miss Clark, who took for her subject the whole question of the entry of women into the medical profession and the progress made in the medical education of women during the past fifty years. This excellent idea for interesting girls who are approaching womanhood in both medicine as a profession and the New Hospital for Women as an institution wherein medicine is practised achieves a double purpose: it will in all probability tend to increase the entry of women medical students in London, and it will also spread amongst the rising generation an interest in institutional work which can only be fraught with good results.

### FRENCH DECORATIONS FOR BRITISH WOMEN.

A correspondent, who signs herself "Royal Red Cross," has sent to the *Morning Post* a strong protest against the old-established regulation that British subjects cannot accept and wear foreign decorations without the expressed consent of His Majesty, which in this connection means the Foreign Office. The grumble arises out of the visit lately paid by the President of the French Republic, M. Poincaré, to a hospital for French soldiers staffed by British medical women and nurses. Owing to the regulation aforesaid, it was impossible for M. le Président to carry out his intention of decorating the "Médecin-Chef," a Scottish lady, and some of her helpers, because the necessary authority from London had not been received. Where the grumbler goes wrong is in her statement that this is a new rule. It is, of course, not new, but very old and familiar, as anyone may see for himself by studying the old files of the *London Gazette*, in which the King's permission to any of his subjects to receive a decoration from a foreign ruler is always published. The French Government are just as well aware of the regulation as anyone else; so if the President was really baulked of his wish, the main reason is that his officials did not allow sufficient margin of time for the securing of the necessary permission, which obviously cannot be given in a moment or without inquiry into the status and antecedents of the person to be decorated, as well as the organisation with which the work has been connected. Still, it is interesting and gratifying to learn that an English medical woman is to receive the Legion of Honour for medical work in France.

### OCTOBER 14, 1916.

Hospital Saturday, which this year is fixed to take place on the above date, is of unusual importance from the fact that the result will show whether or not the enthusiasm, which has led to an increased income in spite of the absence of innumerable subscribers and honorary workers, is a permanent fact or only a transient phase. If figures are good guides the financial position of the Fund is stronger than ever. But the question is, Will this strength remain after the enthusiasm for the war has been succeeded by victory? Will it survive the subtler and searching difficulties which will follow from a declaration of peace through the dislocation of industry, the fear or fact of unemployment, the glut of the labour market, and the fall in wages that may possibly ensue from these conditions? When they occur, as in some measure we fear is likely to be the case, there will be an increased demand for hospital treatment, at the very moment when the Saturday Fund may find it most difficult to maintain its present income. It is therefore essential that the workers on whom the activity of the collections at present depends should be increased as far as possible in number, and encouraged as far as possible by public support, so that the grip of the Fund on the public conscience may not be relaxed, and the organisation of its work may be perfected and maintained to face the stress of hard times ahead. That is why far-sighted people are so anxious that Hospital Saturday this year shall prove a great success, for the stronger the Fund is made in time of war the better equipped will it be to meet the troubles that the end of a great war invariably brings in its train.

### A RED CROSS CONTROVERSY.

AN unfortunate controversy has arisen in Glasgow over the alleged non-fulfilment of the condition laid down by the Highland Association in giving £1,000 towards a ward in Woodside Hospital, to be reserved for Gaelic-speaking soldiers. The Association lately passed a resolution calling for the repayment of its donation on the ground that very few Gaelic-speaking soldiers had been admitted to the ward in question, and that it had not been reserved for them. After an acrimonious correspondence in the newspapers, the War Executive Committee of the Scottish Branch of the Red Cross Society has issued a reply in which they state that they are willing to refund the money if formal application be made for it, but that the Highland Association is wrong in suggesting that a breach of faith has occurred, since in January, and again in July, the Scottish Command had instructed the central hospital to remove all Gaelic-speaking soldiers, who were fit and willing to be transferred, to the Gaelic ward in Woodside Hospital; and, further, that the War Office has been asked to divert such patients at Southampton if possible. The issue really turns not only on the efforts made and the fitness and agreement of the men to be moved, but also on the number of Gaelic-speaking soldiers. How far the Highland Association represents a large body we do not know, but a good case has been made by the Red Cross Society, whose willingness to refund the money, on formal application being made for it, cannot fail to impress the public with the sincerity of efforts in the matter.

### A GOVERNMENT GRANT FOR MEDICINAL HERB GROWING?

THE attempts which have been made in various parts of the country to encourage the growth of medicinal herbs should have taken on a more practical phase as the result of last week's meeting under the Central Committee for National and Patriotic Organisation. The object was to unify the somewhat sporadic efforts of the past few months, through the establishment of a central body by which all existing organisations should be affiliated to each other in a federation, loose enough for local freedom, and close enough for mutual support and the interchange of information. It is hoped by some that the new committee now formed to draft a scheme for the central body will prove strong enough to obtain protection for the growth of such drugs as belladonna, which, it is claimed, cannot be produced on commercial lines unless it is encouraged by a tariff. The alternative or complementary proposal, which was discussed by Sir Sydney Olivier, of the Board of Agriculture, would be for a Government grant in aid of the industry; but he pointed out that this would depend upon the central body possessing a constitution of which the Board of Agriculture would approve. With the practical encouragement thus given there is a chance that the revival of this ancient industry may be maintained, and therefore we trust that the new central body will succeed in affiliating all the herb-growing associations to it.

### A SUGGESTED ALTERATION OF NAME.

FROM the Derby Board of Guardians there has come a suggested alteration in the name of the Boards of Guardians, which is meeting with a considerable amount of support. It is proposed that instead of the designation "Poor-Law Union" now borne by these controlling bodies, the title "Public Services Board" should be substituted as being more appropriate to the character of the work. The new appellation, it is thought, would be calculated to kill existing false prejudice, and admit of the machinery being utilised in a greater degree for national work. Following on this it is maintained that the rate now known as the "Poor Rate" is a misnomer, and misleading to the public, and should be entitled the "Public Services Rate." The work of Boards of Guardians has been considerably widened of recent years, and comprises not only the administration of relief to persons mainly incapacitated by sickness and physical or mental disabilities, but the care of deserted and orphaned children, widows with dependent children, and duties in connection with assessment, the registration of births, deaths, and marriages, vaccination, and the administration of part of the Children Act. Hence there is good ground for the suggested alterations.

### ZEPPELINS AS A SOURCE OF INCOME.

THE damage caused by Zeppelin bombs, to property at least, is being compensated to some extent by the manufacture of rings, charms, and bracelets from the ruins of those Zeppelins which our defensive guns and airplanes are bringing down in satisfactorily increasing numbers. These souvenirs of destroyed Zeppelins, manufactured into trinkets of this kind, are to form one of the chief attractions of the street sales which will be held as part of the collecting machinery on October 19, "Our Day," when the proceeds of sale and collection will be devoted to the funds of the Red Cross Society for the benefit of wounded soldiers. Since economists have declared that in the last analysis demand and supply are discovered to be indistinguishable, no one with a sense of humour should complain if there are as many of these mementoes for sale as the public are ready to buy. The proper attitude is to inquire as to the *bona fides* of the collectors rather than too anxiously into the origin of the materials out of which Zeppelin relics are made. The Red Cross will, of course, guarantee that all the souvenirs sold under its authority are genuinely made from Zeppelin wrecks. So the public should be strictly on its guard against buying these mementoes from any but authorised Red Cross workers. The great thing is to see that the money goes into the Red Cross purse; and we hope that every step has been taken to warn off bogus collectors from the great harvest that will assuredly once again be reaped.

### A CALDOW COT AT THE LONDON HOSPITAL.

The East End Tradesmen's Association in connection with the London Hospital was lately desirous of endowing and naming a cot in that institution in recognition of the forty-four years' service of Mr. J. B. Caldow, who has been treasurer of the Association for that length of time. Mr. Caldow has recently been obliged to give up this splendid lifework of his owing to ill-health. The usual endowment necessary to name a cot at the London Hospital is five hundred guineas; but in view of the special circumstances of Mr. Caldow's case, and the very valuable work which he and the East End Tradesmen's Association have done for the charity, Viscount Knutsford has given his consent, as chairman of the hospital, to the acceptance of two hundred and fifty guineas instead of double that sum. Both the Tradesmen's Association, for its happy thought in desiring to perpetuate the memory of so valuable and so untiring an officer, and Lord Knutsford, in refusing to allow red-tape to stand in the way of realising that desire, are to be congratulated on the endowing of this cot and on the connection with it of so honoured a name as that of Mr. Caldow.

### THE NEW YORK EPIDEMIC OF POLIOMYELITIS.

The latest news from America is to the effect that the exceedingly serious epidemic of anterior poliomyelitis (infantile paralysis), which has devastated New York and the district around (see The Hospital, August 26, 1916, p. 454; and September 9, 1916, p. 539), is showing distinct signs of abating, as was anticipated would be the case when once the hot-weather season was over. By the last week in September the number of new cases had declined to forty per day, a quite sufficiently formidable total; and only about fifteen deaths a day were being notified. There is thus every reason to hope that the pestilence will shortly be stayed; its ravages have been truly terrible, and the roll of its victims must by now reach well into five figures. Great preparations have been made by the authorities to guard against spread of the (as yet unidentified) contagion in the public schools. Prior to the beginning of the new term the teachers went through special courses of instruction for this purpose; and, wisely, poliomyelitis was not the only disease whose prevention it was sought to inculcate. Special arrangements for disinfecting the class-rooms have also been made, with the usual American energy and pushfulness.

### AN OBJECT-LESSON IN FINANCIAL MANAGEMENT.

One of the institutions which necessity has forced to base its income upon a very large annual subscription list is the Bristol Royal Infirmary, and we are glad to see that the large Capern legacy, which has been used to liquidate the once huge capital debt on the new surgical block, has encouraged rather than diminished the number of subscribers. In fact, the net increase of income from this source for the half-year is £210. But since the invested sums are still small, a further £5,000 a year from annual subscriptions is needed if the ordinary income and expenditure are to balance each other. This would be considered a very large order elsewhere, but since nearly £6,000 has been received from this source during the half-year, its further development does not seem impossible among a population which has been educated to regard annual subscriptions as the main resource of the Royal Infirmary, and the principal duty of themselves. This is an object-lesson in what can be done in gaining support from what is generally regarded as the most difficult source of income to foster and maintain.

### A LONG WAITING LIST AT SWANSEA.

The long waiting list was a subject of complaint by one of the members at a meeting of the board of management of the Swansea Hospital. It was suggested that some rearrangement of wards, with the addition of more beds, might be effected. To this, however, Dr. Knight objected. When something of the kind now suggested was attempted previously, he said, the result was the resignation of two members of the honorary medical staff, and they experienced difficulty in getting the resignations withdrawn. Eventually the waiting list question was referred to the house committee for consideration. Grateful acknowledgment was made of the magnificent financial assistance given to the institution by works in the locality, and it was stated that as a result of this aid from the working classes the board would be enabled to carry out some big improvements after the war. The income for last year was £16,615, and the expenditure £11,978; debt on general account was reduced from £4,080 to £655.

### DEATH OF A LIVERPOOL EYE SURGEON.

Liverpool is much the poorer by the death at the Front of Lieutenant-Colonel Arthur Nimmo Walker, R.A.M.C. He was well known by reason of his researches into ophthalmia neonatorum, in which disease he was acknowledged as an expert. Like his father before him, he was intimately associated with the St. Paul's Eye and Ear Hospital, where a memorial ward commemorates his father's splendid work. He was also ophthalmic surgeon to the David Lewis Northern Hospital, surgeon to the School for the Indigent Blind, and assistant lecturer in anatomy in the University of Liverpool. Some years ago, when Dr. Hope, the Liverpool Medical Officer of Health, inaugurated a campaign to check ophthalmia neonatorum, Dr. Walker placed his services and the resources of St. Paul's Hospital at the disposal of the city's medical department. The success which attended the campaign was largely due to this valuable co-operation, and the health committee, bearing that in mind, last week ordered a letter of sympathy to be sent to the late officer's relatives. In medical, as well as in social, circles Lieutenant-Colonel Walker was very popular, and in him the medical and nursing staff at St. Paul's have lost a sincere friend and devoted colleague.

### A YOUNG AND VIGOROUS INSTITUTION.

It is good to know that the Manchester Babies' Hospital has successfully weathered a second annual meeting, and is now explaining the need for increasing the number of its beds from thirty to at least seventy. So many of the disorders of infancy are associated with nutrition, or malnutrition, that the hospital's threefold work of treatment, training (of health visitors), and research is badly needed, and Dr. Niven, the city medical officer of health, bases considerable hopes upon the training provided, which will enable cases not only to be followed up, but also to be detected in their early stages, and thus valuable preventive work to be achieved. Medical students, as well as girl probationers and health visitors, receive training, and it is a good claim to support that was lately made by Mrs. E. D. Simon when she remarked that the Manchester Babies' Hospital was staffed by several doctors with various theories, and was not used as a laboratory for experiment by any one dietician, as she averred to be the case elsewhere. This hospital is one of Manchester's most promising institutions, and we hope that Dr. Niven's statements on its behalf will be received with the attention they deserve, so that the hospital may be quickly extended to cope with the wide field that it has been established to cover.

### OUTPUT IN RELATION TO HOURS OF WORK.

Amongst the dozen interesting memoranda issued by the Health of Munition Workers Committee there has been none of more striking value than the twelfth of the series, just issued as an appendix to Memorandum 5, on hours of work. This appendix is the work of Dr. H. M. Vernon, of Magdalen College, Oxford, and it presents some unique statistics marshalled and arranged with great skill. To appreciate the conclusions reached, the Memorandum, which is eminently readable, should be consulted in the original; but some of them can be quoted within a brief compass. For men engaged in really heavy labour, such as that of sizing fuse bodies, the maximum hours of work consistent with steady output and efficient work, both in quantity and quality, are estimated at 56 or less per week. Men engaged on fairly heavy work can best be employed for 60 or rather more hours a week; while men engaged on comparatively light labour reach their maximum of productiveness when working 70 or even more hours. On the other hand, women employed on what is (for them) moderately heavy labour (though for a man it would be deemed light) do best at 56 hours' work a week; those on light work can usefully do rather more than 60 hours. On very light work women are said to stand even 76 hours fairly well, though Dr. Vernon doubts whether the output is really much better than in a 64-hour week.

### A SUSPICION OF GARROTTING DISPELLED.

An inquest concluded on September 28 at the Southwark Coroner's Court was chiefly remarkable for the removal of the suspicions entertained by a house surgeon of Guy's Hospital that the case might possibly be one of garrotting. A middle-aged woman, in a state of intoxication and severely injured, was found in the darkness by a carter, and was taken by the police to Guy's Hospital, where she died. She had sustained many injuries, including fractures of breast-bone, ribs, jaw, spine, and pelvis, presumably through being run over while lying helpless in the street. There were, however, certain marks on the throat which led the house surgeon, when giving his evidence, to suggest that possibly the woman had been garrotted before she came to be in the roadway. The inquest was adjourned for an expert investigation of this suggestion; and when it was resumed a well-known pathologist gave a reasoned opinion to the effect that the marks in question were simply due to *post-mortem* staining and could not possibly be due to violence of the nature suggested. Although the house surgeon, as it turned out, found a mare's nest, he is at least to be commended for being on the alert to assist the ends of justice and for not being satisfied with the obviously more likely explanation of how the woman came by her injuries.

### WOMEN ON ASYLUM BOARDS.

The desirability of enlisting the co-operation of women in the management of lunatic asylums was emphasised last week by Earl Fortescue presiding over a meeting of the Devon County Council. He pointed out that the Devon County Asylum contains 821 female inmates, but apart from the attendants there is no woman assisting in the management of the institution. His lordship suggested that the Council would be in a better position to deal with the welfare of the female lunatics, and with many other questions lately added to their responsibilities, if a few ladies were appointed as aldermen when the earliest opportunity arose.

### THE MEDICAL SOCIETY'S PLAQUE FROM BOLT COURT.

After the general meeting of the Medical Society of London, to be held on October 9, the Fellows will be invited to inspect the plaque, removed from the Society's old house in Bolt Court, which has now been placed in the library of the Society. This plaque was erected at Bolt Court by Dr. John Coakley Lettsom, the founder of the Medical Society; it was in this court that Dr. Samuel Johnson lived, as well as Dr. Lettsom. Now that the house is to be pulled down, it is obvious that No. 11 Chandos Street is the appropriate resting-place of Lettsom's plaque. The discussions and papers so far announced on the Society's programme for the new session are all connected with military and naval medicine and surgery. The Lettsomian Lectures will be delivered by Colonel Cuthbert Wallace, C.B., and the oration by Sir William Osler, M.D., F.R.S.

